# Dissecting the genetic features and evolution of *Staphylococcus aureus* sequence type 88: a global perspective

**DOI:** 10.1128/msystems.01142-24

**Published:** 2024-11-12

**Authors:** Ye Jin, Chenyang Gao, Gaoqin Teng, Zhenchao Zhou, Wangxiao Zhou, Man Huang

**Affiliations:** 1 Department of General Intensive Care Unit, The Second Affiliated Hospital of Zhejiang University School of Medicine, Hangzhou, Zhejiang, People's Republic of China; 2 Key Laboratory of Early Warning and Intervention of Multiple Organ Failure, China National Ministry of Education, Hangzhou, Zhejiang, People's Republic of China; 3 College of Environmental and Resource Sciences, Zhejiang University, Hangzhou, Zhejiang, People's Republic of China; 4 Clinical Laboratory Center, The Second Affiliated Hospital & Yuying Children’s Hospital of Wenzhou Medical University, Wenzhou, Zhejiang, People's Republic of China; China Agricultural University, Beijing, China

**Keywords:** *Staphylococcus aureus*, sequence type 88, evolutionary dynamics, transmission, φST88-1

## Abstract

*Staphylococcus aureus* sequence type (ST) 88, encompassing both methicillin-resistant *S. aureus* (MRSA) and methicillin-sensitive *S. aureus* (MSSA) phenotypes, is globally prevalent and commonly associated with skin and soft tissue infections. Despite its widespread occurrence, comprehensive genomic studies on this clone remain scarce. In this study, we performed detailed genomic analyses on 130 ST88 isolates derived from severe bloodstream infections alongside 275 publicly available ST88 sequences. Our phylogenetic analysis identified four distinct clades, with evidence suggesting independent evolution and significant clonal expansion of ST88 in China, particularly within clade I, which appeared to have emerged circa 1964. We documented notable interregional, international, and even intercontinental transmission of ST88 isolates. Variability in the distribution of SCC*mec* and *spa* types was observed across clades. Our *in silico* analyses indicated distinct patterns in the distribution of resistance genes, virulence genes, and mobile genetic elements among the clades, with clade I notably harboring the highest prevalence of the intact *sraP* gene and an independently acquired novel prophage, φST88-1. Conversely, clade IV exhibited deletions within the *sasC* gene, with certain sub-clades lacking the *sdrDE* and *fnbB* genes, underscoring the superior adhesive capabilities of clade I. *In vitro* experiments confirmed enhanced biofilm formation in clade I isolates, although the levels of hemolysis and cytotoxicity were similar across clades. Pan-genome-wide association study revealed that core SNPs, rather than the accessory genome, are the primary contributors to the diversification of the ST88 clades. These findings enrich our understanding of the genetic foundations underpinning the transmission dynamics and phenotypic diversity of ST88 clones globally.

**IMPORTANCE:**

Understanding the evolution and transmission of *Staphylococcus aureus* ST88 clones is critically important due to their spread within food, hospital, and community environments, leading to significant health issues. Despite its prevalence, detailed genomic insights into ST88, particularly regarding its diversity and evolutionary dynamics, have been lacking. Our comprehensive genomic analysis of 130 ST88 isolates from severe bloodstream infections, alongside 275 sequences from public databases, significantly advances our understanding of this pathogen. We identified four distinct evolutionary clades, demonstrating the independent evolution and substantial clonal expansion of ST88 in China, as well as its ability to spread across regions and continents. The diversity among the isolates was evident in their unique profiles of *SCCmec* elements, antibiotic resistance genes, virulence genes, and mobile genetic elements. Our findings underscore the critical role of core genomic variations over accessory elements in driving the diversification of ST88. This enhanced understanding provides new insights that could inform more effective control strategies, crucial for developing interventions to combat the global spread of this formidable pathogen.

## INTRODUCTION

*Staphylococcus aureus* (*S. aureus*) is a clinically significant Gram-positive pathogen, with approximately 25% to 30% of healthy individuals persistently colonized, primarily in the anterior nares, groin, oropharynx, perineum, and axillae (1–3). The pathogenicity of *S. aureus* is largely attributed to its production of various virulence factors, including extracellular toxins, extracellular enzymes, and surface protein adhesins (4). These factors enable methicillin-resistant *S. aureus* (MRSA) to cause a range of infections, from relatively minor skin and soft tissue infections (SSTIs) to severe, life-threatening conditions, such as pneumonia, osteomyelitis, and endocarditis, occurring in both community-acquired and hospital-acquired settings (5).

*S. aureus* remains a globally prevalent pathogen, with its presence documented across hospitals, communities, and agricultural settings. Extensive molecular epidemiological studies have shown greater genetic diversity among MSSA isolates compared with MRSA isolates within specific regions (6–8). Globally, the dominant MRSA lineages include ST1, ST5, ST8, ST22, ST59, and ST239, with ST239, ST5, and ST59 particularly prominent in several Asian countries (9, 10).

While the ST88 lineage of both MRSA and methicillin-sensitive *S. aureus* (MSSA) is relatively rare, it is globally distributed and has been identified in clinical specimens from various Asian countries, including Bangladesh, Myanmar, and Nepal (11, 12). In China, ST88 has been frequently detected in animal-derived foods and associated with human SSTIs (13–15). Recent studies suggest that ST88, particularly in MRSA form, may be a potent biofilm producer, complicating antimicrobial treatment (16). However, most research on ST88 has focused on epidemiological aspects, with limited in-depth analysis of its virulence characteristics and genomic features, especially in bloodstream infections (BSIs).

Therefore, while these studies have provided critical insights into the epidemiology of *S. aureus*, particularly MRSA, there remains a gap in understanding the genomic evolution and virulence characteristics of the globally prevalent clones like ST88. This study extends previous epidemiological insights by providing a comprehensive analysis of 130 ST88 isolates from BSIs across 17 provinces in China. Through genomic and phenotypic characterizations, we aim to uncover the genomic evolution, potential transmission patterns, and clonal expansion of ST88 in China, while comparing these findings with global ST88 isolates. Notably, we have identified a novel prophage, φST88-1, which lacks virulence or resistance genes, offering new insights into the evolutionary dynamics of this lineage. By comparing key traits, such as virulence genes, resistance genes, and mobile genetic elements (MGEs), across different clades and analyzing pangenome repertoires and core single nucleotide polymorphisms (SNPs), this study provides a detailed understanding of the genetic makeup and evolutionary trajectory of ST88, contributing essential knowledge for combating MRSA infections.

## RESULTS

### Geographic distribution and phylogenetic analysis of global *S. aureus* ST88 clones

**Fig 1:**
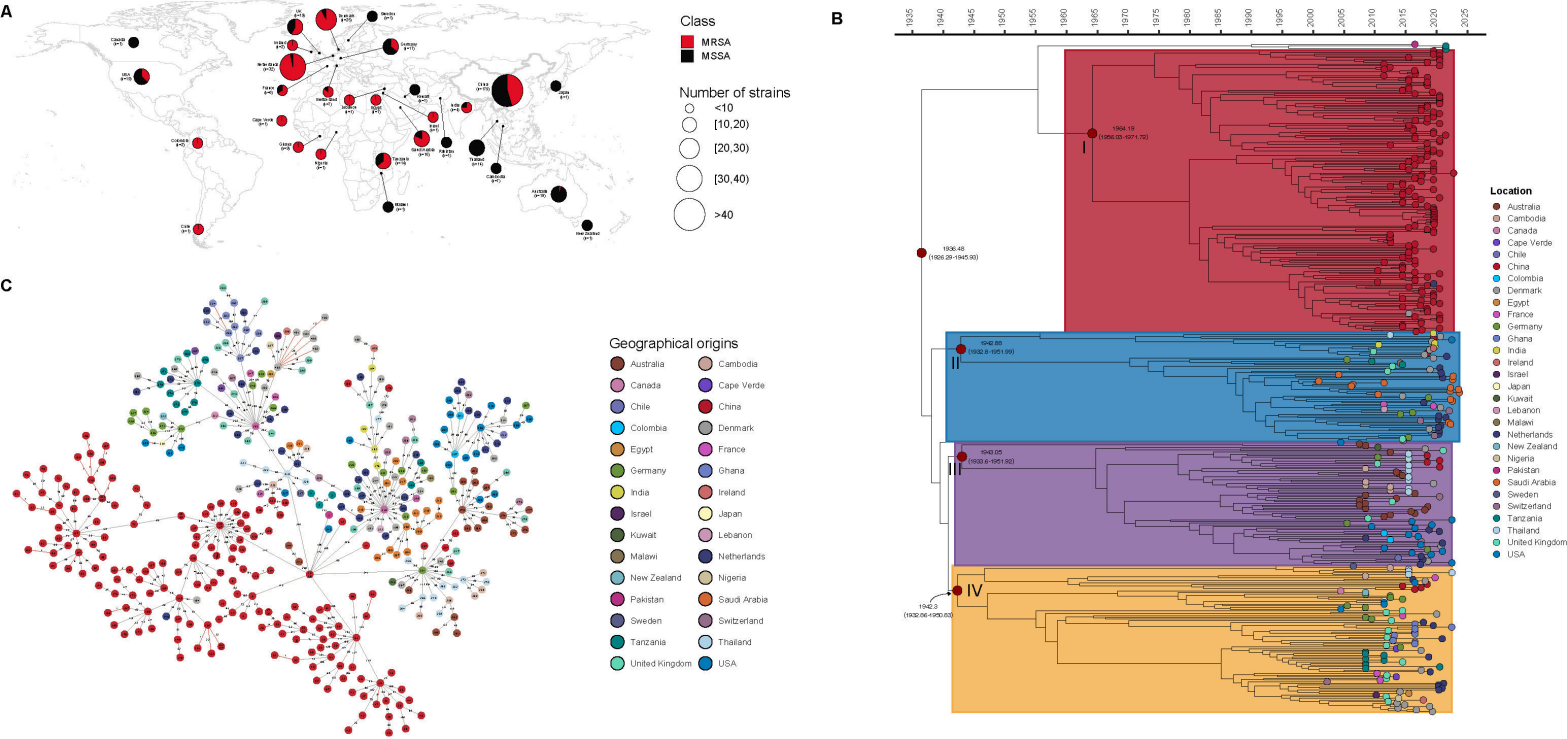
Relationships among 405 ST88 isolates in this study. (**A**) Geographic distribution of the isolates, with MRSA and MSSA indicated by red and black partitions within each circle, respectively. The sizes of the circles correspond to the number of strains present at each location. (**B**) Time-calibrated phylogenetic tree of the 405 ST88 isolates, with tip colors representing the geographic origin of the strains. The divergence times and 95% highest posterior density (HPD) intervals for each clade are indicated at the respective nodes. (**C**) Minimum spanning tree (MST) of the ST88 isolates, where each circle denotes an isolate, colored according to geographic origin. The numbers along the connecting lines denote the number of SNPs in pairwise comparisons. Transmission clusters are highlighted with a red dotted line, defined by a threshold of 23 SNPs.

To investigate the molecular evolution of the ST88 strains in this study, we analyzed the genomes of 130 ST88 *S. aureus* isolates collected by our own strain collection along with an additional 275 publicly available ST88 genomes sampled between 1998 and 2023. The geographic distribution of ST88 is shown in Fig. 1A, encompassing samples from 30 countries, including China (*n* = 179), New Zealand (*n* = 32), Denmark (*n* = 25), the United Kingdom (*n* = 19), Australia (*n* = 19), and the United States (*n* = 19), with representation from Asia, Europe, the Americas, Australia, and Africa.

**Fig 2:**
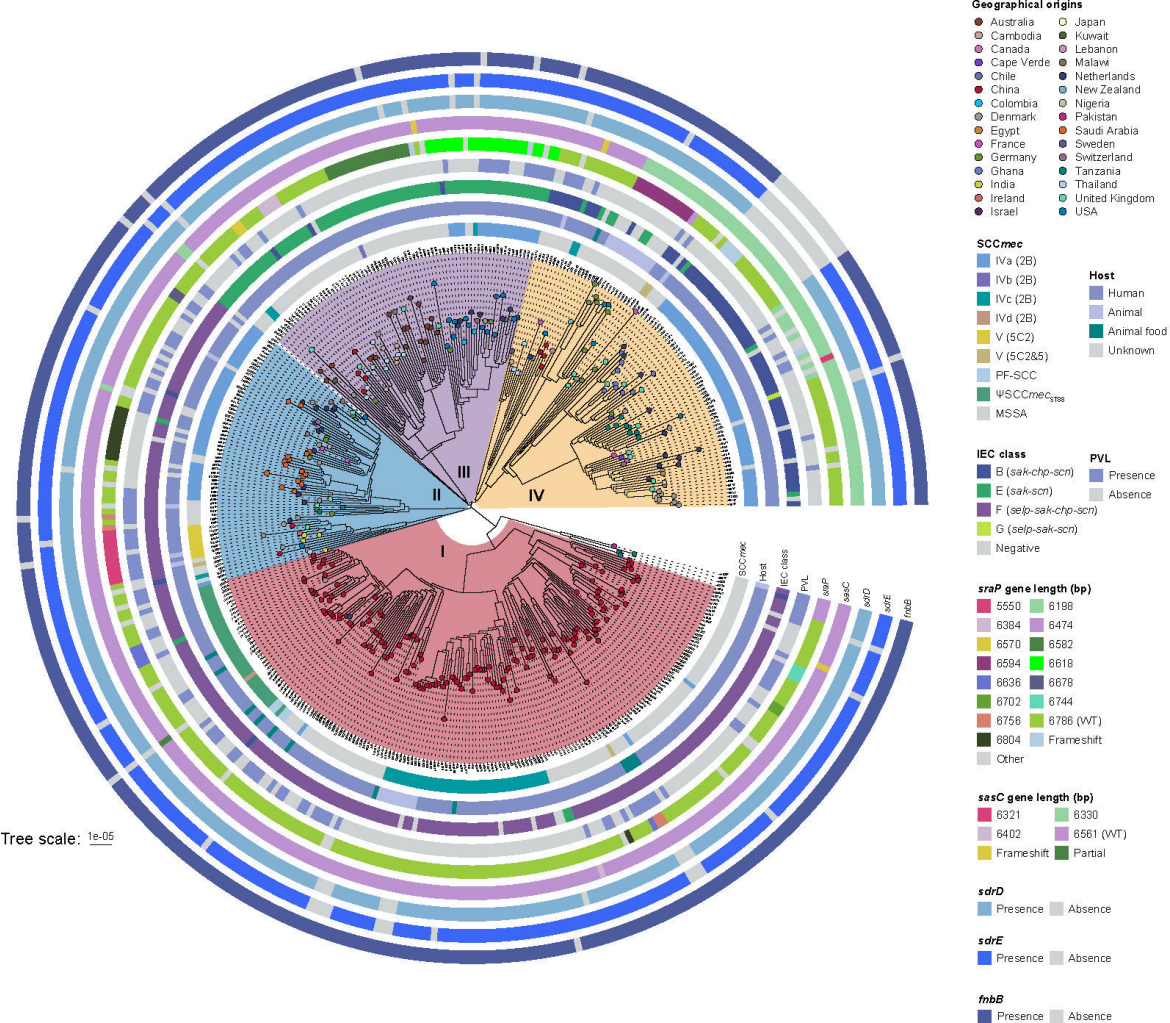
Phylogenetic structure of 405 ST88 strains. Attributes including *SCCmec* type, host type, IEC class, presence of PVL genes, sequence lengths of *sraP* and *sasC*, and presence of *sdrD*, *sdrE*, and *fnbB* are annotated on the tree, displayed from the innermost to the outermost circle. The *spa* types for each isolate are indicated in parentheses adjacent to the isolate names. ND, not determined.

The 130 ST88 strains from China (MSSA: 78 strains; MRSA: 52 strains) were isolated from BSI patients across 17 provinces (Fig. S1), with the highest number of strains originating from Zhejiang Province (*n* = 36), followed by Anhui Province (*n* = 33), Xinjiang Province (*n* = 11), Fujian Province (*n* = 10), Yunnan Province (*n* = 8), and Shandong Province (*n* = 7). Detailed information on these ST88 strains is listed in Table S1.

To analyze the evolutionary position and genetic diversity of the ST88 isolates, we identified SNPs within the core genomes of 405 ST88 isolates. All ST88 strains are divided into four major phylogenetic clades (I to IV, Fig. 1B and 2), with a common ancestor dating back to approximately 1936.48 (95% highest posterior density [HPD] interval: 1926.29 to 1945.93). The divergence time of clades II, III, and IV within the ST88 lineage occurred more closely: clade II emerged in 1942, clade III in 1943, and clade IV in 1942. In contrast, clade I, with 98.83% of strains from China, diverged the latest, in 1964. These findings suggest that the expansion of ST88 in China occurred over several decades following its initial emergence. Additionally, Chinese ST88 clones were also distributed among clades II, III, and IV: except for one MRSA strain in clade II; all other strains were MSSA. These results indicate that although Chinese ST88 clones originated from various lineages, they primarily clustered into a distinct lineage, clade I.

Spa typing identified 111 known *spa* types among the 405 ST88 strains. Notably, the isolates in clade I exhibited high diversity, including 43 different *spa* types, with t3622 (12.28%, 21/171), t1376 (11.70%, 20/171), and t2310 (10.53%, 18/171) being the most prevalent. Further, the primary types of SCC*mec* elements carried by ST88-MRSA strains differed between clade I and other clades. Clade I exhibits the highest diversity, containing six different types of *SCCmec* elements, with the predominant types being SCC*mec* IVc (23.39%, 40/171) and the pseudo-*SCCmec* element ΨSCC*mec*_ST88_ (19.30%, 33/171). Notably, all strains harboring the ΨSCC*mec*_ST88_ belonged to clade I. In contrast, ST88-MRSA strains in clades II, III, and IV predominantly carried SCC*mec* IVa, with prevalence rates of 65.15%, 25.68%, and 59.55%, respectively. These findings suggest that ST88-MRSA strains from China can independently acquire multiple types of SCC*mec* elements.

Additionally, while some closely related strains from the same country clustered together, we observed clustering of strains from different countries or different provinces within China (Fig. 2; Fig. S1A), suggesting the possibility of international and inter-regional transmission within China. In fact, the phylogenetic tree even revealed evidence of intercontinental transmission of ST88 isolates. Specifically, clade I showed transmission between strains from China, Denmark, and the Netherlands. Similarly, clade IV indicated transmission between food samples from Japan and the United States. Further, we determined the number of SNPs among the isolates to better identify potential transmission pairs and conduct epidemiological investigations. As shown in Fig. 1C; Fig. S1C, the number of SNPs among the ST88 isolates ranged from 0 to 436. In contrast, the SNPs within the ST88 clone, as represented by the red dashed lines, were all fewer than 24, indicating a closer genetic relationship among these strains and suggesting up to 79 transmission pairs. These findings suggest that ST88 isolates may be spreading across different geographic regions.

### Comparison of virulence genes in ST88 clades

**Fig 3:**
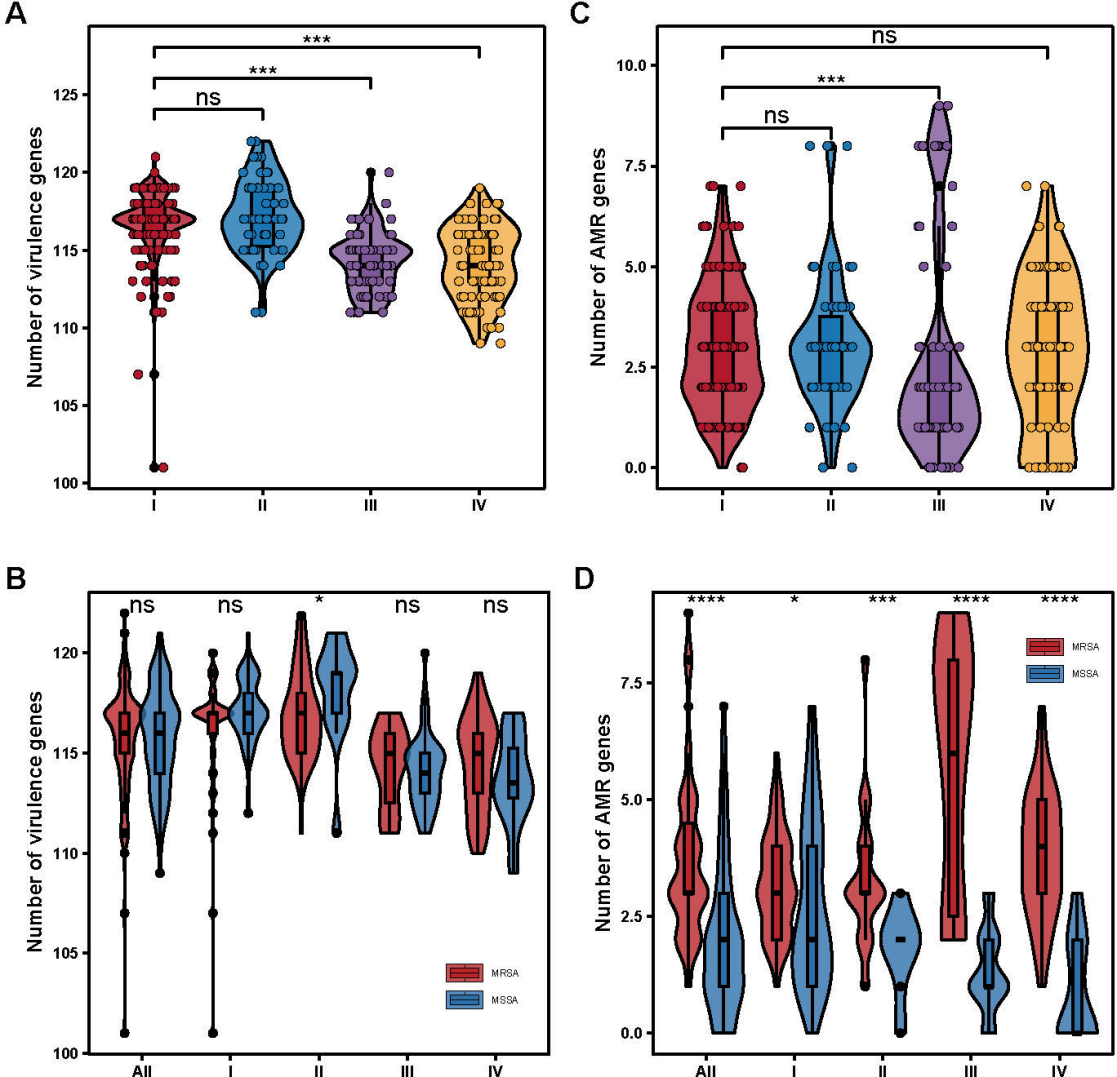
Distribution of virulence and AMR genes in ST88 isolates. (**A**) Distribution of virulence genes across isolates from various clades. (**B**) Comparison of virulence gene counts in MRSA versus MSSA isolates from different clades. (**C**) Distribution of acquired AMR genes across isolates from various clades. (**D**) Comparison of acquired AMR gene counts in MRSA versus MSSA isolates from different clades. *, *P* < 0.05; ***, *P* < 0.001; ****, *P* < 0.0001. ns, no significant difference between groups.

Next, we conducted a detailed comparative analysis of the virulence genes among the ST88 clades. As shown in Fig. 3A, there were no significant differences in the virulence gene profiles between ST88 isolates from clades I and II. However, significant differences in the number of virulence genes were observed between clade I and clades III and IV.

**Fig 4:**
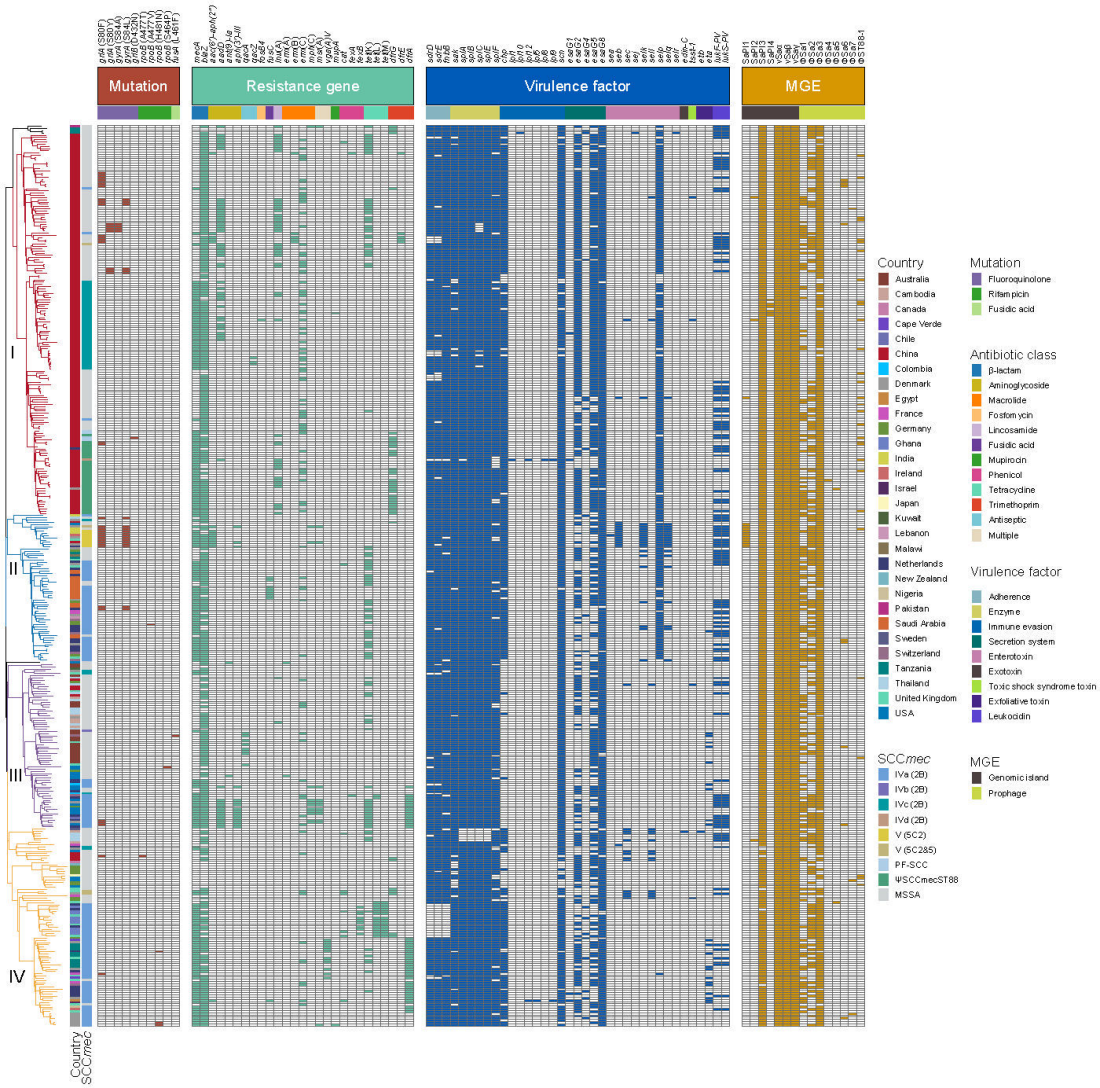
Distribution of chromosomal mutations, AMR genes, virulence genes, and mobile genetic elements (MGEs) among 130 ST88 isolates from China and 273 ST88 isolates from global sources. Virulence genes are categorized into nine classes: adherence, enzyme, immune evasion, secretion system, enterotoxin, exotoxin, toxic shock syndrome toxin, exfoliative toxin, and leukocidin. AMR genes are grouped into 12 categories: β-lactam, aminoglycoside, macrolide, fosfomycin, lincosamide, fusidic acid, mupirocin, phenicol, tetracycline, trimethoprim, antiseptic, and multiple resistance. The presence of each trait is indicated by color-coded boxes corresponding to the respective category. Genes with a prevalence exceeding 95% across all four evolutionary clades were excluded from the figure to emphasize the distribution of differentially prevalent virulence genes.

Most isolates within each clade (≥95% of isolates per clade) harbored virulence genes related to adhesion, immune evasion, type VII secretion system, extracellular enzymes, hemolysins, phenol-soluble modulins, exotoxins (*set*), leukocidins (*lukD* and *lukE*), and iron acquisition. Specifically, as shown in Fig. 4, the Panton–Valentine leukocidin (PVL) genes *lukF*/*PV* and *lukS*/*PV* were dispersed across all clades. Moreover, almost all ST88 strains carried the immune evasion cluster (IEC) genes (93.8%, 380/405), with four IEC types B (*sak-chp-scn*, *n* = 71), E (*sak-scn*, *n* = 85), F (*selp-sak-chp-scn*, *n* = 223), and G (*selp-sak-scn*, *n* = 1) identified. However, the distribution of IEC types varied significantly across clades, demonstrating clade-specific genetic patterns: in clades I and II, most ST88 strains were of the IEC F type, with prevalence of 92.98% (159/171) and 92.42% (61/66), respectively. In contrast, type E dominated in clade III, encompassing 94.59% (70/74) of its strains, while 71.91% (64/89) of clade IV strains were characterized by type B. In addition, the toxic shock syndrome toxin gene *tsst-1* and the exfoliative toxin gene *eta* were detected in only 4 and 22 ST88 isolates, respectively. Furthermore, we found that methicillin resistance in ST88 strains did not significantly impact the overall virulence gene profile (Fig. 3B and 4). The pathogenic potential, as inferred from the virulence factors, remained consistent across both ST88-MSSA and ST88-MRSA strains. This highlights the importance of including both methicillin-sensitive and methicillin-resistant strains in analysis to fully understand the epidemiology and pathogenicity of the ST88 lineage.

Notably, different clades revealed significant variations in the sequences of the *sraP* gene, associated with bacterial adhesion. Structural analysis of the wild-type SraP domain in ST88 strains revealed that it possesses a serine-rich repeat protein (SRRP) consisting of an N-terminal signal peptide, a short serine-rich region (SRR1, 91–244 aa), a non-repeat region (NRR, 245–751 aa) that functions as the ligand-binding domain, a long serine-rich region (SRR2, 752–2211 aa), and a C-terminal cell wall anchoring motif (CW, 2212–2261 aa). The wild-type *sraP* gene (6,786 bp in length) was found at a higher proportion in clade I (86.55%, 148/171) compared with other clades (<60%, *P* < 0.05, Fig. 2). As shown in Fig. S2A, compared with clade I strains, other clades exhibited significant deletions in the SRR2 region of the *sraP* gene. Moreover, clade IV also showed 77 amino acid internal deletions within the *sasC* gene, spanning from the latter portion of DUF1542-7 domain to the early portion of DUF1542-8 domain (Fig. S2B). Additionally, certain sub-clades of clade IV were found to lack the *sdrDE* and *fnbB* genes (Fig. 2). These genetic variations suggest that the adhesive capacity of clade I strains might be higher than that of other clades, while the adhesive capacity of clade IV strains might be reduced.

To validate the potential role of these adhesins in clade I, we conducted biofilm formation assays on all 130 ST88 isolates using crystal violet staining in 96-well plates. The results, as shown in Table 1, indicated that isolates from clade I exhibited significant biofilm-forming capacity, with approximately 63.1% (*n* = 77) of these isolates forming biofilms of varying intensities. Notably, 11.5% (*n* = 14) of clade I strains demonstrated particularly strong biofilm-forming ability. In contrast, isolates from clades II to IV did not form any detectable biofilms. These findings suggest that the enhanced biofilm formation observed in clade I may be associated with the presence of specific surface adhesins, such as *sraP*, *sdrDE*, and *fnbB*, which were predominantly found in clade I isolates, thereby reinforcing the distinct adhesive potential of this clade.

**TABLE 1:** Comparison of biofilm formation ability among ST88 clade I to clade IV strains in this study^*a*^

Biofilm formation	Total (*n* = 130)	Clade I (*n* = 122)	Clade II (*n* = 2)	Clade III (*n* = 4)	Clade IV (*n* = 2)
Weak, *n* (%)	25 (19.2)	25 (20.5)	0	0	0
Moderate, *n* (%)	38 (29.2)	38 (31.1)	0	0	0
Strong, *n* (%)	14 (10.8)	14 (11.5)	0	0	0
None	53 (40.8)	45 (36.9)	2 (100)	4 (100)	2 (100)

^*a*^ Optical density cut-off (ODc) = average OD of negative control + 3× standard deviation (SD) of negative control. Weak, OD ≤ 2ODc; moderate, 2ODc < OD ≤ 4ODc; strong, OD >4ODc.

To validate the virulence potential suggested by our genomic analysis, we conducted hemolysis and macrophage infection assays on representative isolates from each clade. As shown in Fig. S3, the hemolysis assays demonstrated no significant differences in hemolytic activity among the ST88 isolates across all clades, indicating uniformity in this aspect of virulence. Furthermore, macrophage infection assays using RAW264.7 cells revealed that isolates from clade I induced inflammatory cytokine expression (TNF-α and IL-6) at levels comparable to those observed in isolates from other clades. These findings suggest that, although clade I exhibited enhanced biofilm formation, the cytotoxic effects on immune cells remain consistent across the different clades, highlighting a conserved aspect of virulence in ST88 strains.

### Antibiotic susceptibility of Chinese ST88 lineages

We also assessed the susceptibility of 130 ST88 isolates from China to 16 commonly used antibiotics, with detailed results presented in Table S2. No resistance to linezolid, vancomycin, or daptomycin was detected among the isolates, consistent with the predicted genotypes. Rifampicin exhibited the best antimicrobial activity, with 99.2% of the isolates being sensitive, while penicillin showed the poorest activity, with a resistance rate of 96.9%. One rifampicin-resistant ST88 isolate harbored the *rpoB* H481N mutation. Additionally, all ST88 strains exhibited a low resistance rate to fluoroquinolones (10.0%, *n* = 10), and we identified mutations in the *grlA* and *gyrA* genes that may contribute to fluoroquinolone resistance. Specifically, mutations, such as *grlA* (S80F/S80Y), *gyrA* (S84A/S84L), and *grlB* (D432N), were present in most quinolone-resistant ST88 strains. Moreover, all MRSA isolates carried the *mecA* gene and were resistant to oxacillin. These data suggest a strong correlation between the presence of resistance genes and the observed antimicrobial susceptibility patterns in these isolates. This finding underscores the utility of genomic sequencing as a valuable tool for clinicians in managing complex *S. aureus* infections.

To understand the varying resistance patterns among global ST88 strains, we assessed the presence of antimicrobial resistance (AMR) genes. There were no significant differences in the number of resistance genes among clades I, II, and IV (Fig. 3C). Importantly, resistance in ST88 was primarily acquired through horizontal gene transfer, with point mutations being relatively rare (e.g., *grlA*, *ropB*, and *fusA*) (Fig. 4). Mutations in *grlA* are known for its role in fluoroquinolone resistance, mutations in *rpoB* are associated with rifampicin resistance, and mutations in *fusA*, linked to fusidic acid resistance, represent crucial factors contributing to AMR in certain isolates. However, in our study, such mutations were infrequent.

Strains in clade I harbored specific resistance genes, particularly *dfrG*, which encodes a dihydrofolate reductase enzyme conferring resistance to trimethoprim, with a prevalence of 16.37%. In contrast, clades III and IV were characterized by the presence of the *dfrA* gene, which also encodes a trimethoprim-resistant dihydrofolate reductase, with prevalence rates of 21.62% and 35.96%, respectively.

Additionally, over 27% of clade I strains carried the *aadD* and *lnu*(A) genes, with prevalence rates of 27.49% and 29.24%, respectively. The *aadD* gene confers resistance to aminoglycosides, while *lnu(A)* encodes a lincosamide nucleotidyltransferase, conferring resistance to lincosamides. Interestingly, these resistance genes were less common in other clades, highlighting the distinct AMR profiles across the different clades. Therefore, this genetic diversity underscores the importance of considering both point mutations and horizontally acquired resistance genes when analyzing the AMR characteristics of ST88 isolates. As shown in Fig. 3D, the number of AMR genes in ST88 MRSA strains was significantly higher than in ST88 MSSA strains across all clades, particularly in clades II, III, and IV (*P* < 0.001).

### Characterization of the large MGEs in ST88 clones

We then analyzed the distribution of pathogenicity islands and prophages within the ST88 isolates, as these large MGEs are crucial components of the *S. aureus* genome and could significantly influence the evolution of virulence. Our results indicated that all isolates in each clade harbored three pathogenicity islands: vSaα, vSaβ, and vSaγ. Additionally, over 93% and 98% of the isolates carried a prophage φSa3 and SaPI3 (Fig. 4), respectively, which were identified as the hotspots for recombination (Fig. S4).

The comparative genomic analysis of φSa3 sequences carried by clades I/II and III/IV revealed significant differences across several functional modules (Fig. S2C). Specifically, except for the lysis module, the sequence variations were observed in the IEC encoding module, the structural modules, encompassing the packaging, head and tail regions, the DNA replication/transcription regulation module, and the lysogeny module. These findings underscore the genetic diversity and evolutionary dynamics of φSa3 prophage within different clades of ST88, highlighting the potential impact of these variations on phage biology and the interaction with their bacterial hosts.

**Fig 5:**
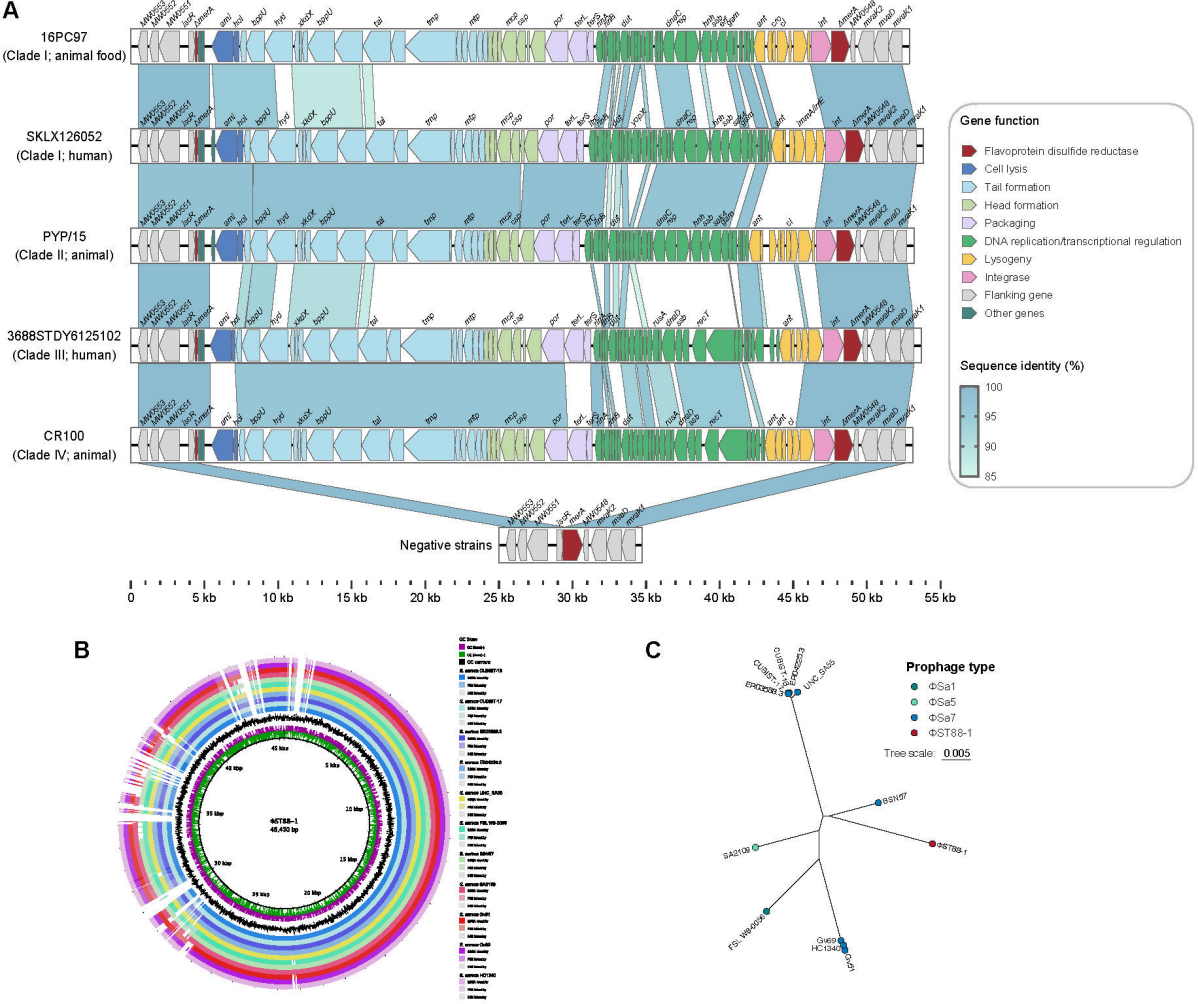
Schematic representation of φST88-1 and its comparison with similar prophages. (**A**) Comparison of φST88-1 among ST88 strains from each clade. Genes within the prophage are represented by arrowed boxes, which are colored according to their functional classification. (**B**) Circular representation of the φST88-1 and comparative genomic analysis with other similar prophages. Circles 1–11 refer (from outer to inner circle) to homologous regions of selected *S. aureus* relative to the φST88-1. Circles 12–13 represent GC content and GC skew of the φST88-1, respectively. (**C**) Phylogenetic analysis of the φST88-1 with other similar prophages, with tip colors representing the prophage types carried by the strains.

Further, within clade I, a novel, uncharacterized prophage lacking resistance or virulence genes was discovered. This prophage, designated φST88-1, was sporadically distributed throughout the entire clade (Fig. 4), suggesting that despite its low prevalence, clade I isolates might have independently acquired this prophage, reflecting similar adaptive signatures. Structural analysis of φST88-1 revealed that it comprised genes associated with several modules, including the lysis module, the structural module (tail, head, and packaging), the DNA replication/transcription regulation module, and the lysogeny module. Genetic context analysis showed that φST88-1 was integrated into the *merA* gene, which encodes flavoprotein disulfide reductase. Recent studies have shown that the *merA* gene encodes the hypothiocyanous acid (HOSCN) reductase MerA, conferring resistance to neutrophil oxidants, such as HOSCN and hypochlorous acid (HOCl) (17, 18). Therefore, strains carrying φST88-1 may have a reduced ability to withstand oxidative stress compared with those without the prophage. Sequence differences between φST88-1 from human-origin isolates in clade I and animal-origin isolates in clade II, as well as φST88 from human-origin isolates in clade III and animal-origin isolates in clade IV, were primarily concentrated in the DNA replication/transcription regulation and the lysogeny modules. This suggests potential transmission of φST88-1 between animals and humans, possibly accompanied by recombination events during evolution (Fig. 5A). BLAST searches under the NCBI nucleotide database revealed that φST88-1 shared over 95% identity and 80% coverage with prophages found on 11 *S*. *aureus* chromosomes. However, even the five most similar prophages (located in *S. aureus* strains CUBIST-18, CUBIST-17, ER03588.3, ER04225.3, and UNC_SA55) only covered 83% of the φST88-1 genome (Fig. 5B). Phylogenetic analysis suggests that φST88-1 and φSa7 may have originated from a common ancestor and undergone recombination during their evolutionary process (Fig. 5C).

### Core SNPs rather than the accessory genome significantly contribute to the diversification of ST88 clades

**Fig 6:**
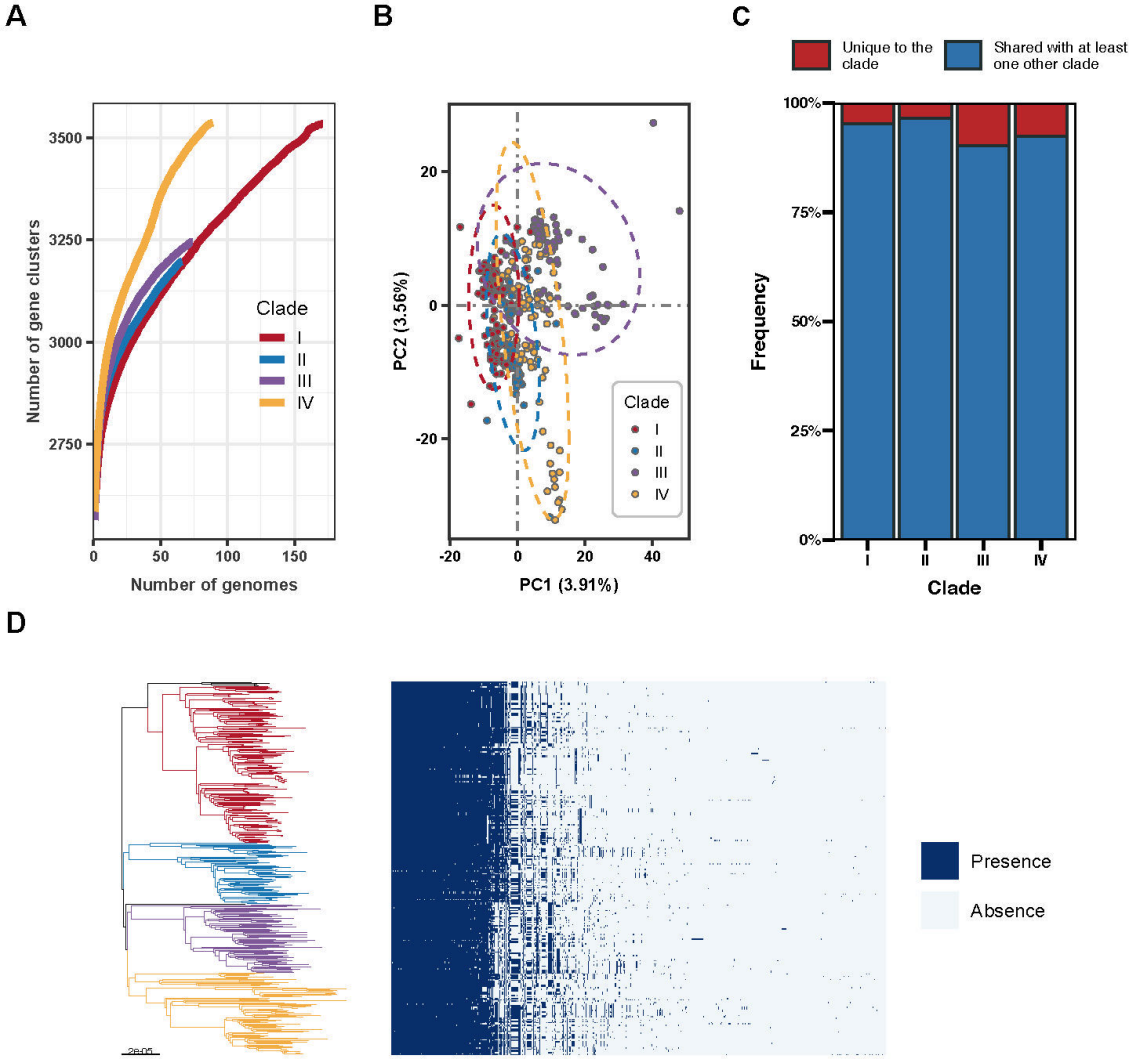
Pan-genome diversity of ST88 isolates. (**A**) The accumulation curve illustrates the relationship between the pan-genome size and the number of genomes across the four major clades, with the curve colors differentiated by clade. (**B**) PCA based on the accessory gene content matrix of 405 isolates, where each circle represents an isolate, colored according to its clade. (**C**) The frequency of pan-genome composition across different clades. The red area represents the proportion of genes specific to a single clade (i.e., absent in other clades), while the blue area shows the proportion of genes shared with at least one other clade. (**D**) Difference of accessory genome between different clades of the ST88 lineage. The heatmap provides the presence and absence (see legend) of genes ordered as per the core phylogeny.

To further explore the genomic differences between ST88 strains from various clades, we analyzed the complete gene repertoire of all isolates, encompassing both core and accessory genes (i.e., the pangenome). Our findings revealed that the pangenome of global ST88 strains comprised 4270 unique genes, with accessory genes accounting for 54.10%. As shown in the pangenome accumulation curve (Fig. 6A), the rate of new gene accumulation in clade IV strains was faster than in other clades, indicating higher pangenome diversity in these ST88 strains. However, the pangenomes of all clades exhibited an open state, suggesting ongoing gene acquisition.

Principal component analysis (PCA) was performed based on the accessory gene content matrix to investigate genetic composition differences between and within clades. The results indicated that, rather than showing discrete, clade-specific clustering, most strains within each clade clustered together in the principal component coordinates, indicating a high similarity in the accessory genome profiles across different clades (Fig. 6B). Similarly, as shown in Fig. 6C, after excluding clade-specific core genes, clades I, II, III and IV only had 4.69%, 3.46%, 9.81%, and 7.60% of their genes classified as clade-specific, respectively. Analysis through the genome-wide association study (GWAS) identified a total of 1, 27, and 1 clade-specific gene associated with clades II, III and IV, respectively, with all clade-specific genes in clade III being components of the prophage φSa3 (Table S3). Overall, the genetic differences among the clades were minimal, with gene diversity accumulating at the population level rather than the clade level (Fig. 6D).

However, as shown in Fig. S5A, the PCA based on the core SNP matrix revealed discrete, clade-specific clustering, further underscoring that the evolution of the different clades might be primarily driven by clade-specific SNP differences. Further analysis based on GWAS detected a total of 39, 54, and 17 clade-specific SNPs associated with clades I, II and IV, respectively (Table S5). Interestingly, three clade I-specific nonsynonymous SNPs were related to bacterial adhesion (*sasC*), iron uptake (*sirA*), and virulence regulation (*rsp*); additionally, one clade II-specific nonsynonymous SNP was involved in leukocidin (*lukD*). Upon categorizing genes with clade-specific nonsynonymous SNPs according to COG categories, we found that in clade I, genes were enriched in categories “transport and metabolism of inorganic and carbohydrate” and “transcription”; genes in clade II showed enrichment in categories “transport and metabolism of inorganic, lipid, amino acid and carbohydrate”; and genes in clade IV predominantly belonged to categories, such as “translation, ribosomal structure, and biogenesis,” “signal transduction mechanisms,” ”transcription,” and “amino acid transport and metabolism.”

## DISCUSSION

In recent years, the field of whole-genome sequencing (WGS) has advanced rapidly, accompanied by a substantial reduction in costs. This has significantly expanded the use of WGS in the study of bacterial pathogens, thereby deepening our understanding of bacterial evolution (19, 20). Notably, several studies have reconstructed the evolutionary histories of major pathogenic clones of *S. aureus*, providing new insights into their emergence, expansion, and dissemination, as well as monitoring the dynamic changes and evolutionary processes of MRSA epidemiology. For instance, the evolutionary history of the highly successful CA-MRSA CC80 clone in Europe has been systematically reconstructed using phylogeography and molecular clock analysis (21). This research suggested that resistance to fusidic acid might have driven the widespread dissemination of this lineage. Additionally, Hsu and colleagues pioneered the use of phylogenetic analysis to investigate the dynamic competition between MRSA ST22 and ST239 clones within the Singapore healthcare system (22). These studies highlight the critical role of WGS in tracking and understanding the evolutionary trajectories and epidemiological patterns of bacterial pathogens, thereby aiding in the development of more effective control and prevention strategies.

ST88 is commonly detected in both humans and food animals (23). Studies have reported that ST88 accounts for 8.8% to 16.3% of CA-MRSA infections in Chinese children (24, 25), and it is frequently associated with SSTIs (15). Comprehensive understanding of the epidemiology and transmission of specific clones is crucial for controlling MRSA infections. However, there is a lack of research on the genomic characteristics and evolutionary history of ST88 strains. To address this gap, we collected 130 ST88 isolates from China between 2011 and 2020, along with 275 globally available ST88 isolates, and performed WGS and comparative genomics analyses.

The construction of the global phylogenetic tree distributed the ST88 isolates from China across all four major clades, with 94.41% in clade I, highlighting the high genetic diversity among Chinese strains. Further phylogenomic analysis revealed evidence of frequent transmission events, including interregional transmission within China and inter-country or even intercontinental dissemination of ST88 isolates. Specifically, clinical strains within clade I demonstrated transmission events between China, Denmark, and the Netherlands, suggesting there may be frequent exchanges of people or other vectors among these countries, facilitating the spread of ST88 strains. Similarly, clade IV indicated transmission between food samples from Japan and the United States. This further implies that ST88 isolates may spread not only through human contact but also *via* the food supply chain across countries. These findings have significant public health implications. Firstly, the evidence of interregional and international transmission underscores the importance of international collaboration in controlling the spread of ST88. Countries should strengthen surveillance systems to promptly detect and trace the transmission routes of MRSA. Secondly, it is crucial to develop targeted control measures based on the epidemiological characteristics of ST88 in different geographic regions. For instance, enhancing the inspection of imported and exported food could reduce the risk of foodborne transmission. Moreover, our study indicates that although there is genetic diversity among ST88 strains from different geographic regions, they share common evolutionary characteristics. This suggests that ST88 strains have a strong adaptability and transmission capability on a global scale.

Our study also provides comprehensive insights into the genomic diversity and evolutionary dynamics of ST88-MRSA strains, particularly focusing on the distinct characteristics of clade I. Most isolates within clade I, derived from China, were notable for their distinct SCC*mec* element profile compared with strains within other clades, displaying the highest diversity. The predominant types were SCC*mec* IVc (23.39%) and the pseudo-SCC*mec* element ΨSCC*mec*_ST88_ (19.30%). This ΨSCC*mec*_ST88_ element contains the *mecA* gene complex C2 and a series of genes related to heavy metal resistance but lacks an approximately 28 kb region that includes the *ccr* complex (26). These findings suggest a unique ability of ST88-MRSA strains from China to independently acquire a diverse range of *SCCmec* elements. The diversity of *SCCmec* elements in clade I highlights the adaptive potential and genetic plasticity of ST88-MRSA strains from China. This genetic diversity could be a result of various evolutionary pressures and horizontal gene transfer events, enabling these strains to acquire and maintain multiple *SCCmec* elements. The presence of ΨSCC*mec*_ST88_ exclusively in clade I suggests a localized evolutionary event or selective pressure within this geographic region. Moreover, the spread of ST88-MRSA clones across different clades indicates multiple independent acquisitions of methicillin resistance, which could have significant implications for infection control and treatment strategies. The presence of strains with different SCC*mec* types in clades other than clade I further support the hypothesis of distinct evolutionary origins and adaptive pathways for ST88 clones in China.

The observed sequence differences in the *sraP* gene among the clades likely have significant implications for the pathogenicity and host interactions of ST88 strains, as the *sraP* gene encodes a surface-exposed serine-rich repeat glycoprotein (SRRP) in *S. aureus*, playing a crucial role in promoting adhesion and invasion into host epithelial cells (27). The integrity of the SRRP structure in clade I, especially the high prevalence of the intact SRR2 region, suggests that ST88 lineages prevalent in China might possess enhanced adhesive and colonization capabilities, providing a potential selective advantage. These findings highlight the crucial role of the *sraP* gene and related adhesion factors like *sasC* in the evolutionary adaptation and pathogenicity of ST88 strains. Further research is essential to explore the functional implications of these genetic differences and their impact on the epidemiology and clinical outcomes of ST88 infections. Understanding the molecular mechanisms underlying these variations could inform the development of targeted interventions and therapeutic strategies to mitigate the spread and impact of ST88.

Prophages, as large MGEs, are typically repressed and integrated into the host chromosome, often encoding virulence factors, such as superantigens, enterotoxins, PVL, and biofilm formation components (28, 29). These MGEs have significant clinical relevance (30). In this study, we identified a novel, uncharacterized prophage, designated φST88-1, which was integrated into the *merA* gene. This gene functions as a hypothiocyanous acid reductase in *S. aureus*, crucial for defending against this innate immune-derived oxidant (17). Although φST88-1 lacked known resistance or virulence genes, it was found across different isolates in clade I, suggesting that these isolates may have independently acquired this prophage through convergent evolution. This scenario might potentially facilitate the local expansion of clade I strains in China. Notably, the sequence differences between human- and animal-derived φST88-1 suggested that φST88-1 may be transmitted between animals and humans and has undergone recombination events during evolution. These findings highlight the complexity and dynamic nature of prophage integration and evolution within *S. aureus* ST88. The presence of φST88-1 in human and animal isolates across different clades underscores the potential for cross-species transmission and the role of recombination in shaping the genetic diversity of prophages. Future studies are needed to elucidate the functional implications of φST88-1 integration and its impact on the adaptability and pathogenicity of *S. aureus* ST88.

The concept of the pangenome, first introduced by Tettelin et al. in 2005 (31), is defined as the entire set of genomic information within a species. This comprehensive genetic repertoire undergoes dynamic changes through gene loss, gene duplication, and horizontal gene transfer, which can result in varying environmental adaptations among strains of the same species. In this study, our pan-genomic analysis revealed a high level of pan-genomic diversity within ST88 strains. The pangenomes of all clades exhibited an open state, indicating ongoing gene acquisition. PCA demonstrated no significant differences in accessory gene composition between clades, suggesting that genetic diversity accumulates at the population level rather than the clade level, with only minor genetic differences observed between clades. On the contrary, evolution within different clades was primarily driven by clade-specific SNPs. The observed pan-genomic diversity and clade-specific SNP variations underscore the complex adaptive strategies and evolutionary dynamics of ST88 strains. Future research should focus on the functional implications of these clade-specific SNP and their role in the adaptability and pathogenicity of ST88, providing insights into the mechanisms underlying the evolution and spread of this lineage.

While this study offers significant insights into the ST88 lineage of *S. aureus*, particularly regarding its genomic characteristics, virulence factors, and resistance profiles, several limitations must be acknowledged. Although our study primarily focused on genomic analyses, the phenotypic validation was limited. While we conducted biofilm formation, hemolysis assays, and cytotoxicity tests, we did not include other *in vitro* assays, such as invasion or adhesion tests, which could have provided a more comprehensive understanding of ST88’s pathogenic potential and interactions with host cells. Additionally, the limited number of isolates from other clades in this study may affect the representativeness of our experimental results. Furthermore, since the isolates analyzed in this study were predominantly sourced from China, this may not fully capture the global diversity of ST88 strains. While comparisons were made with global isolates, regional variations in strain characteristics and resistance profiles may not have been comprehensively addressed. Also, the absence of detailed patient demographic and clinical history data limits our ability to correlate specific host factors, such as age or comorbidities, with ST88 infections, which could have yielded important insights into ST88’s clinical behavior. Moreover, although we identified and characterized the novel prophage φST88-1, no *in vitro* experiments were conducted to evaluate its impact on the host strain’s fitness, virulence, and resistance. Further experimental work, such as planktonic and biofilm competition assays, is needed to fully elucidate the functional consequences of φST88-1 integration. Preliminary findings regarding φST88-1’s impact on resistance and fitness also require further validation through detailed functional analyses, particularly under oxidative stress and other environmental conditions. Finally, while the study identified correlations between genomic features, such as the presence of the *sraP* gene and biofilm formation, it did not establish causative relationships. Experimental validations are necessary to confirm how these genomic features influence ST88’s virulence.

In conclusion, this study represents the first comprehensive genomic characterization of global ST88 clones and those ST88 strains isolated from BSIs in China. Our findings indicate that Chinese ST88 lineage may have evolved independently across different clades, with significant clonal expansion observed particularly in clade I. We have demonstrated the capacity of ST88 isolates for interregional, international, and even intercontinental transmission. Notably, clade I strains exhibited a distinct SCC*mec* element profile and a higher diversity of *spa* types compared with strains from other clades. Additionally, differences in virulence genes and MGEs were observed among isolates from each phylogenetic clade. For instance, clade I strains showed a high prevalence of the intact *sraP* gene and had independently acquired the novel prophage φST88-1, potentially offering a selective advantage. Importantly, our data strongly support the view that core SNP variations are a significant driving mechanism in bacterial evolution.

## MATERIALS AND METHODS

### Collection of *S. aureus* ST88 isolates

From January 2011 to December 2020, we isolated 3,848 *S*. *aureus* strains from patients with BSI across 22 provinces in China. The test strains were identified using biochemical methods and MALDI-TOF MS (Bruker, Bremen, Germany). Multilocus sequence typing (MLST) was performed on all 3,848 *S*. *aureus* isolates by sequencing seven housekeeping genes (*arcC*, *aroE*, *gmk*, *glpF*, *tpi*, *yqiL*, and *pta*). The sequences of the housekeeping genes were then submitted to the typing database to assign ST (https://pubmlst.org/bigsdb?db=pubmlst_saureus_seqdef). Ultimately, we identified 130 ST88 isolates from all the *S. aureus* isolates (Table S1).

### Antimicrobial susceptibility testing

Following the guidelines outlined by the Clinical and Laboratory Standards Institute (CLSI, 2020), the agar dilution method was employed to assess the susceptibility of the isolates to a panel of 16 antimicrobial agents. These agents included oxacillin, penicillin G, sulfamethoxazole–trimethoprim, ciprofloxacin, levofloxacin, moxifloxacin, gentamicin, amikacin, erythromycin, clindamycin, tetracycline, tigecycline, rifampin, vancomycin, linezolid, and daptomycin. For quality control purposes, *S. aureus* strains ATCC 25923 and ATCC 29213 were utilized as reference strains.

### Whole-genome sequencing and genomic analysis of ST88 isolates

Whole-genome sequencing of the 130 ST88 isolates from our collection was performed on the HiSeq X Ten platform (Illumina, San Diego, CA, USA) with 2 × 150 bp read lengths. The sequencing data underwent assembly after adapter trimming and quality filtering (Phred quality score ≥20) using SPAdes v3.14.1 (32), with initial quality control facilitated by fastp v0.20.1 (33). Annotations of the assemblies were completed using DFAST-core v1.2.11 (34). SCC*mec* and *spa* typing of the ST88 isolates was verified through the web-based tools SCC*mec*Finder (https://cge.food.dtu.dk/services/SCCmecFinder) and SpaFinder (https://cge.food.dtu.dk/services/spaTyper). The pan-genome analysis of ST88 *S. aureus* was conducted using Panaroo v1.3.0 (35), with the core genome defined as genes present in 100% of the isolates, and the accessory genome as genes present in less than 100% of the isolates. PCA of the accessory gene content matrix for the ST88 strains was performed using the prcomp function in R. Additionally, 275 ST88 genomic assemblies, available with geographic locations and sampling dates, were retrieved from the NCBI GenBank database for comparative analysis (Table S1).

### Phylogenetic analysis of ST88 isolates

Alignment of 405 ST88 genomes against the reference genome AUS0325 (GenBank accession no. NZ_LT615218.1) was conducted using Snippy v4.6.0 (https://github.com/tseemann/snippy), which facilitated the generation of a core-genome SNP alignment. Regions undergoing recombination were identified through Gubbins v2.4.1 (36), and the resulting 18,377 recombination-free core-genome SNPs were employed to construct a maximum-likelihood phylogeny using RAxML v8.2.12 (37). This analysis was performed using a GTRGAMMAX model, complemented by 1,000 bootstrap replicates to validate the phylogenetic support. Recombination events among the bacterial strains were visualized using the RCandy v1.0.0 package (38) within R. The temporal origin of the ST88 population was estimated utilizing BactDating v1.1 (39). The computational procedure entailed running the Markov chain Monte Carlo simulations for 100 million cycles, with the initial 50% of each chain discarded as burn-in. Convergence of the model was assured by confirming that effective sample sizes for all parameters exceeded 200. Minimum spanning trees (MSTs) based on core SNP data were constructed using PHYLOViZ v2.0 (https://online.phyloviz.net/index). The PCA based on the core SNP matrix was conducted using the prcomp function in R.

### Comparison of virulence genes, resistance genes, and MGEs among ST88 isolates

Virulence factors and resistance genes were identified utilizing ABRicate v1.0.0 (https://github.com/tseemann/abricate), employing the VFDB and ResFinder databases with minimum thresholds of 85% identity and 85% query coverage, respectively. PointFinder v3.2 (40) was employed to detect antibiotic resistance determinants resulting from chromosomal mutations, requiring a minimum of 90% identity and 90% query coverage for confirmation. Additionally, pathogenicity islands and prophages were detected using BLASTN (https://blast.ncbi.nlm.nih.gov/Blast.cgi) with 85% identity and 85% query coverage, where sequences from pathogenicity islands and prophages of *S. aureus* published on public sequence databases served as reference queries. The schematic representations of MGEs were generated using the genoplotR package of R software (41).

### Biofilm formation

In our biofilm assay, biofilm formation was quantified using the crystal violet staining method in 96-well plates, following the protocol described by Jin et al. (42). Briefly, isolates were incubated in TSB medium (Sigma-Aldrich, St. Louis, MO, USA) supplemented with 0.5% glucose at 37°C for 24 h. After washing with phosphate-buffered saline (PBS, Thermo Fisher Scientific, Waltham, MA, USA), the adhered biofilms were fixed with 95% methanol, stained with 1% crystal violet for 10 min, and then dissolved in 33% glacial acetic acid (Sigma-Aldrich, St. Louis, MO, USA). the optical density (OD) of each well was measured at 492 nm. The OD cutoff (ODc) was calculated as the mean OD of the negative control plus three standard deviations. Strains were classified based on their OD values compared with the ODc as follows: non-biofilm producers (OD ≤ODc), weak biofilm producers (ODc <OD ≤ 2×ODc), moderate biofilm producers (2 × ODc < OD ≤4 × ODc), and strong biofilm producers (OD >4 × ODc), according to the criteria used in previous studies (43).

### Murine macrophage RAW264.7 cytotoxicity assay

Murine macrophage RAW264.7 cells were cultured in high-glucose DMEM (Gibco, Thermo Fisher Scientific, Waltham, MA, USA), supplemented with 10% fetal bovine serum (FBS, Sigma-Aldrich, St. Louis, MO, USA) and 1% penicillin–streptomycin (Thermo Fisher Scientific, Waltham, MA, USA). Cells were seeded into sterile six-well plates (Corning, New York, NY, USA) at a density of 3 × 10⁵ cells/mL and incubated at 37°C in a humidified 5% CO₂ atmosphere for 24 h. We randomly selected four strains from clade I (strain IDs: SKLX52179, SKLX61912, SKLX125907, and SKLX144909), all strains from clade II (two strains; strain IDs: SKLX109324 and SKLX80737), all four strains from clade III (strain IDs: SKLX86304, SKLX120657, SKLX147642, and SKLX79434), and both strains from clade IV (strain IDs: SKLX58966 and SKLX60801) for the cell-based experiments.

Bacterial suspensions were prepared by adjusting the concentration of bacteria to 1.0 × 10⁹ CFU/mL using sterile PBS. A 10-µL aliquot of the bacterial suspension was added to each well, ensuring a bacteria-to-cell ratio of 30:1, resulting in a multiplicity of infection (MOI) of 30. The cells were co-incubated with the bacteria at 37°C for 6 h. Following the incubation, the cell supernatant was collected and centrifuged at 2,000×*g* for 20 min at 4°C to remove debris. The collected supernatant was then used to perform cytokine measurements using enzyme-linked immunosorbent assays (ELISAs) following the manufacturer’s instructions. Cytokine levels were quantified based on the standard provided with the kit (44).

### Hemolytic activity assay

Hemolytic activity was evaluated following a previously described protocol (45). Briefly, strains were cultured in TSB at 37°C for 6 h. Post-incubation, the cultures were centrifuged, and the supernatants were collected. Rabbit red blood cells (RRBC, Thermo Fisher Scientific, Waltham, MA, USA) were washed three times with PBS (pH 7.2). A portion of the supernatant was then combined with the RRBC, and the mixture was incubated at 37°C for 3 h. Triton X-100 (1%, Sigma-Aldrich, St. Louis, MO, USA) was used as a positive control, while RRBC in PBS served as a negative control.

### Detection of clade-specific genes and SNPs

The genes and SNPs associated with isolates from each clade were identified using Scoary v1.6.16 (46), with a significance threshold of *P* < 1e-10 and both sensitivity and specificity greater than 75%. EggNOG-mapper v1.0.3 was used to assign genes to COG categories by searching against the Eggnog database (47).

### Statistical analysis

All statistical analyses were performed using GraphPad (San Diego, CA, USA). Data are presented as mean ± standard deviation (SD). Differences between clades were assessed using the Kruskal–Wallis test. *P* < 0.05 was considered statistically significant. The *P*-values for the expression of inflammatory cytokines TNF-α and IL-6, and for hemolytic activity among the ST88 isolates across all clades were 0.17, 0.59, and 0.23, respectively.

## Data Availability

The whole-genome sequences of 130 ST88 *S. aureus* isolates have been deposited in the GenBank database under BioProject accession no. PRJNA749878.

## SUPPLEMENTAL MATERIAL

Fig. S1 msystems.01142-24-s0001.pdf Relationships among 130 ST88 isolates from China in our own collection.

Fig. S2 msystems.01142-24-s0002.pdf Comparison of *sraP*, *sasC*, and φSa3 between ST88 strains in each clade.

Fig. S3 msystems.01142-24-s0003.pdf Comparison of virulence among different clades of ST88 strains.

Fig. S4 msystems.01142-24-s0004.pdf Recombination plot of the ST88 genomes compared with the reference genome (AUS0325).

Fig. S5 msystems.01142-24-s0005.pdf Core SNP diversity among ST88 clades.

Legends msystems.01142-24-s0006.docx Fig. S1 to S5 legends.

Table S1 msystems.01142-24-s0007.xlsx Information on 405 ST88 isolates.

Table S2 msystems.01142-24-s0008.xlsx Antimicrobial susceptibility of 130 ST88 isolates from China.

Table S3 msystems.01142-24-s0009.xlsx Clade-specific genes as identified by Scoary.

Table S4 msystems.01142-24-s0010.xlsx Clade-specific SNPs as identified by Scoary.

ASM does not own the copyrights to Supplemental Material that may be linked to, or accessed through, an article. The authors have granted ASM a non-exclusive, world-wide license to publish the Supplemental Material files. Please contact the corresponding author directly for reuse.
