# Supplementary figures and images for: Dissecting the genetic features and evolution of Staphylococcus aureus sequence type 88: a global perspective
Source: mSystems. 2024 Nov 12;9(12):e01142-24. doi: 10.1128/msystems.01142-24 (PMC11651095; doi:10.1128/msystems.01142-24)

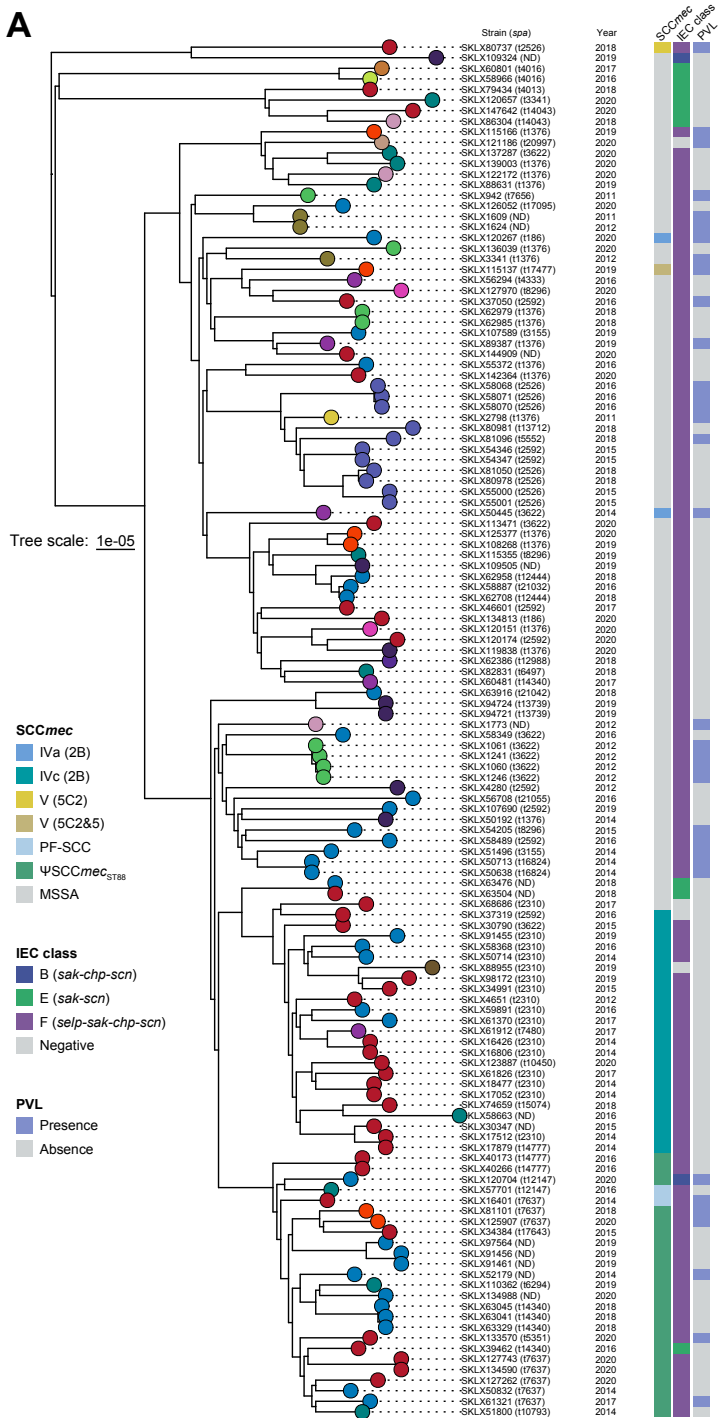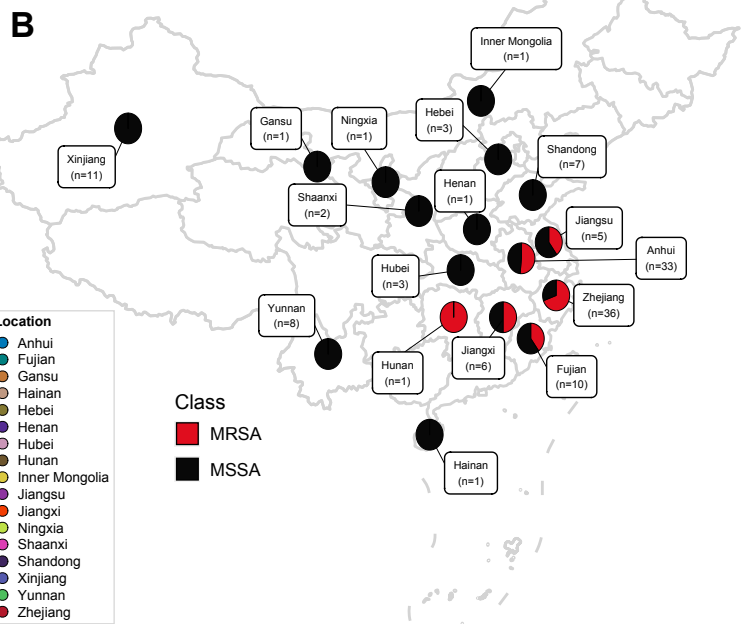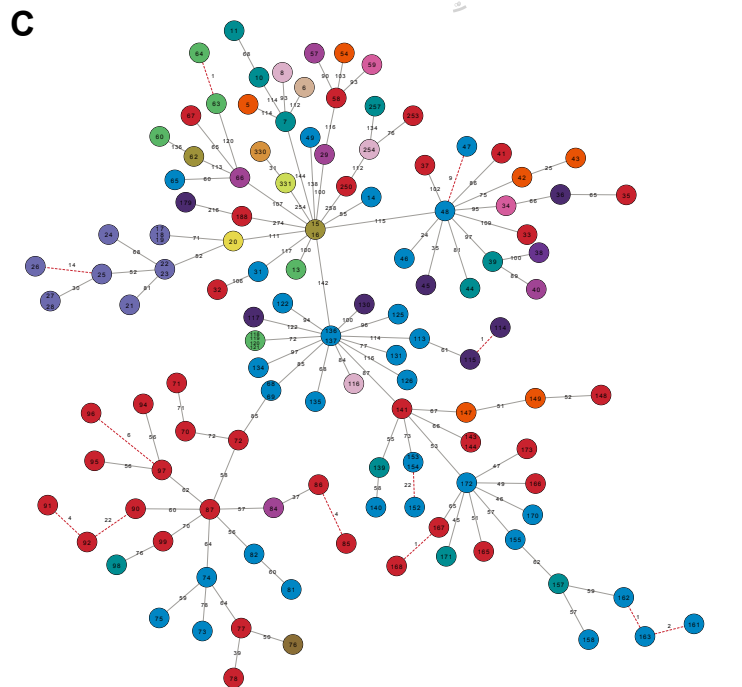

Supplement: Fig. S1 — Relationships among 130 ST88 isolates from China in our own collection. [file msystems.01142-24-s0001.pdf]

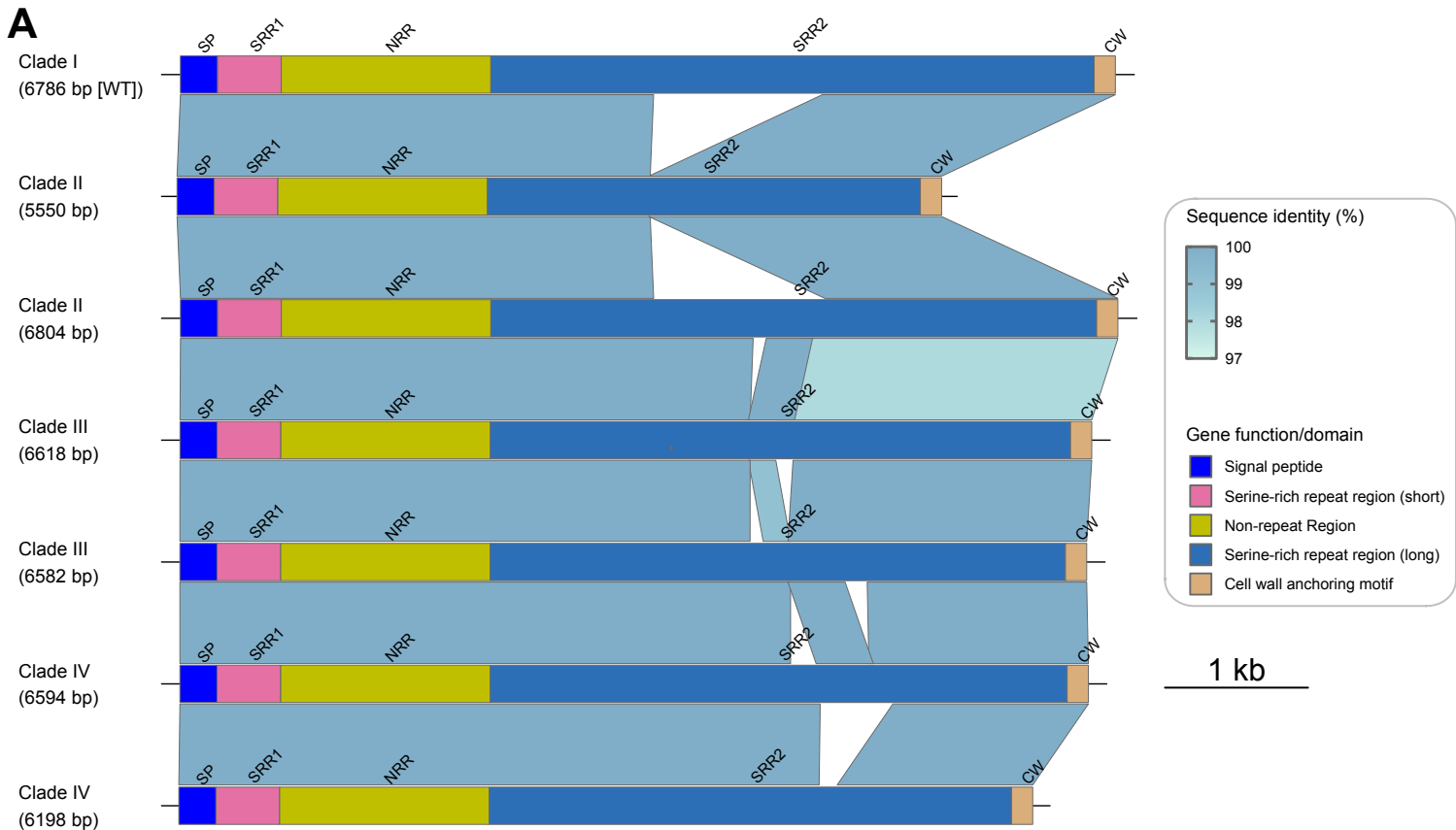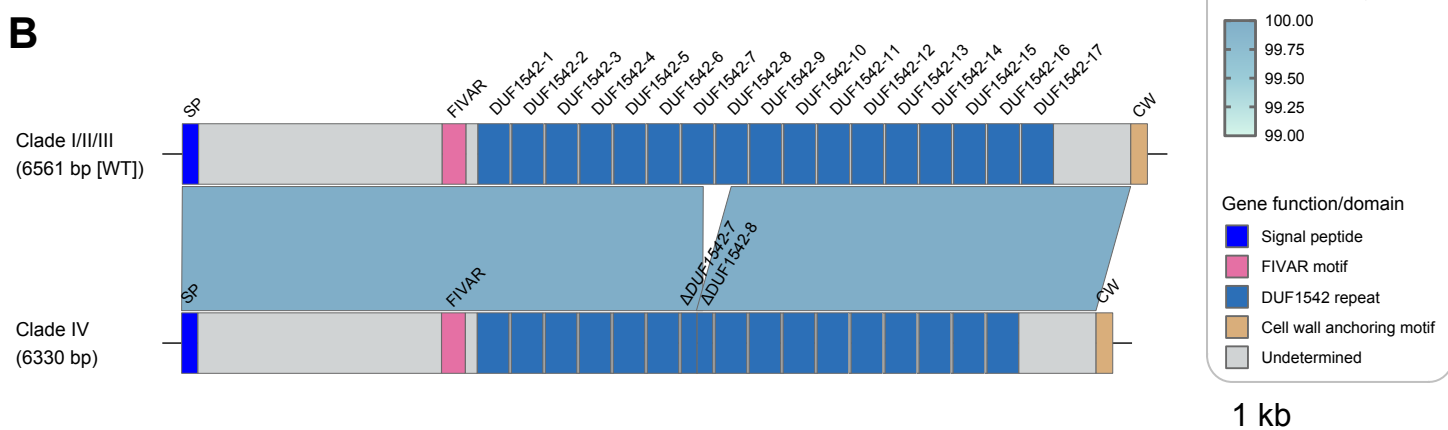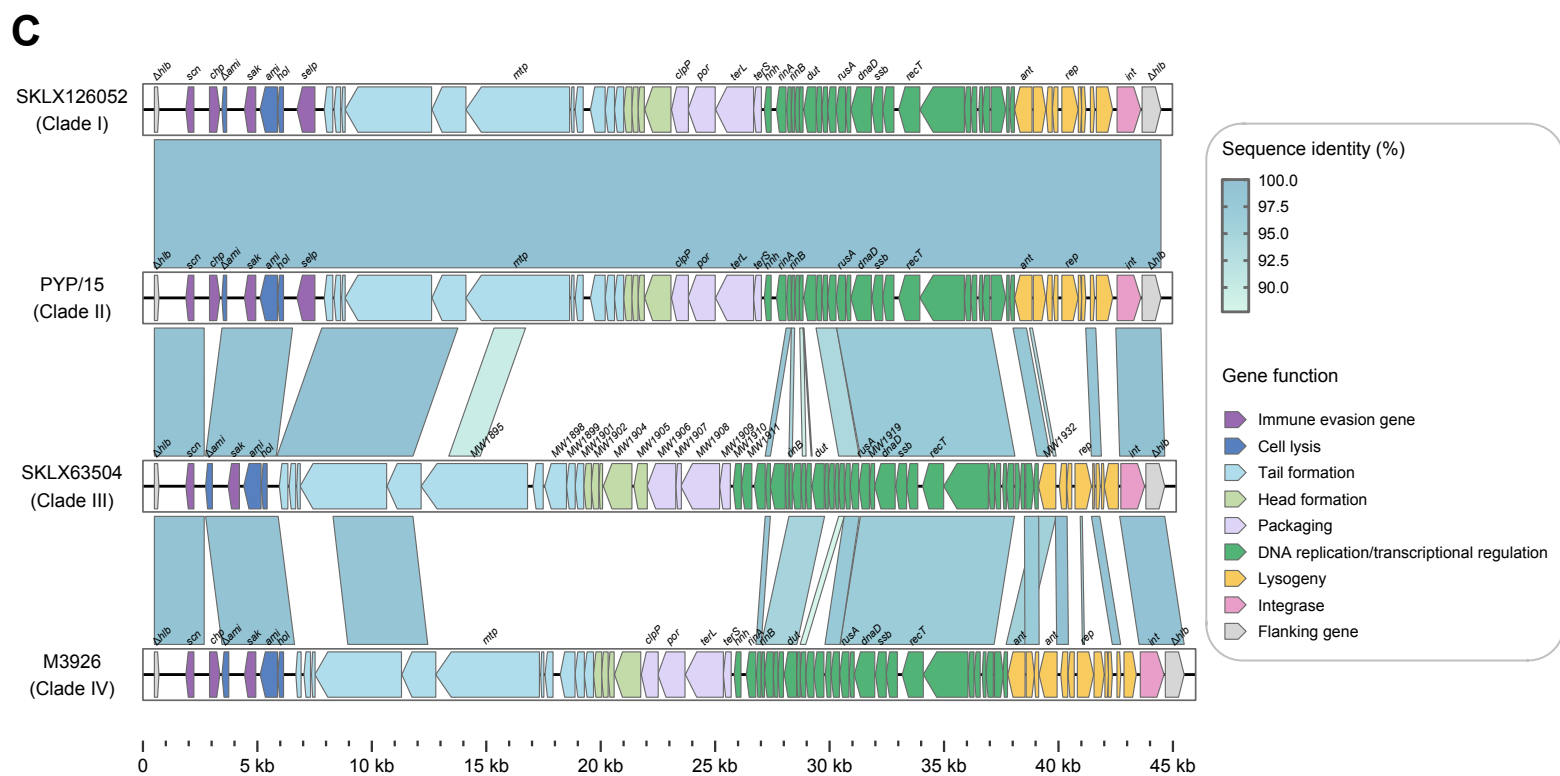

Supplement: Fig. S2 — Comparison of sraP, sasC, and φSa3 between ST88 strains in each clade. [file msystems.01142-24-s0002.pdf]

A

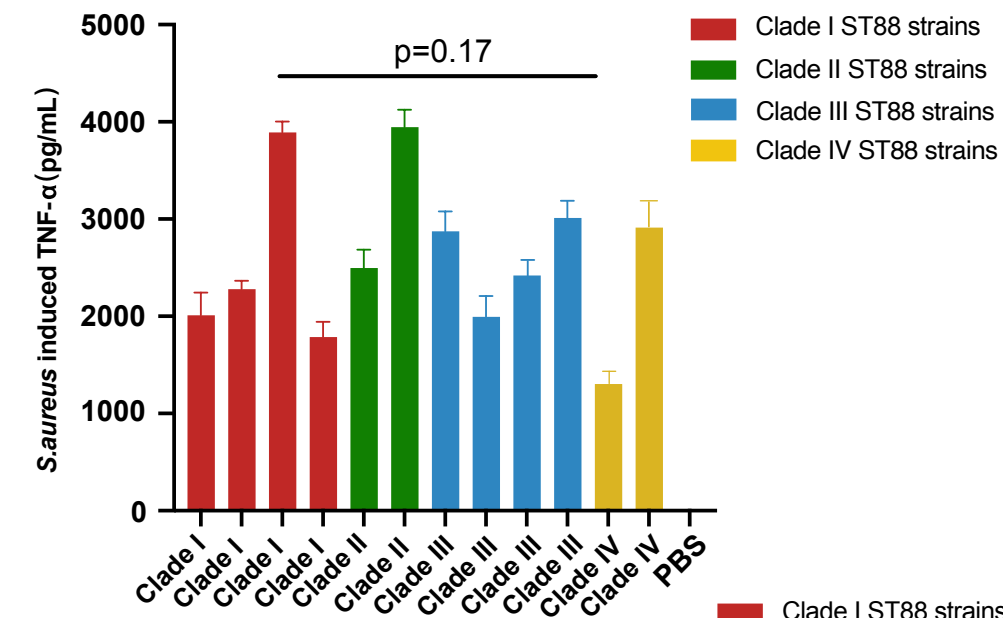

B

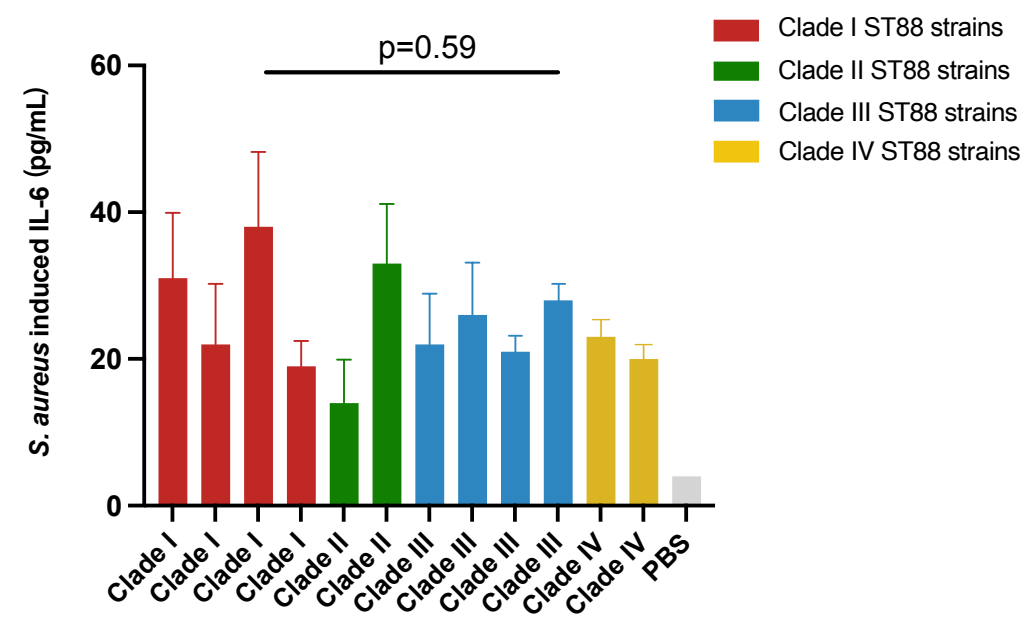

C

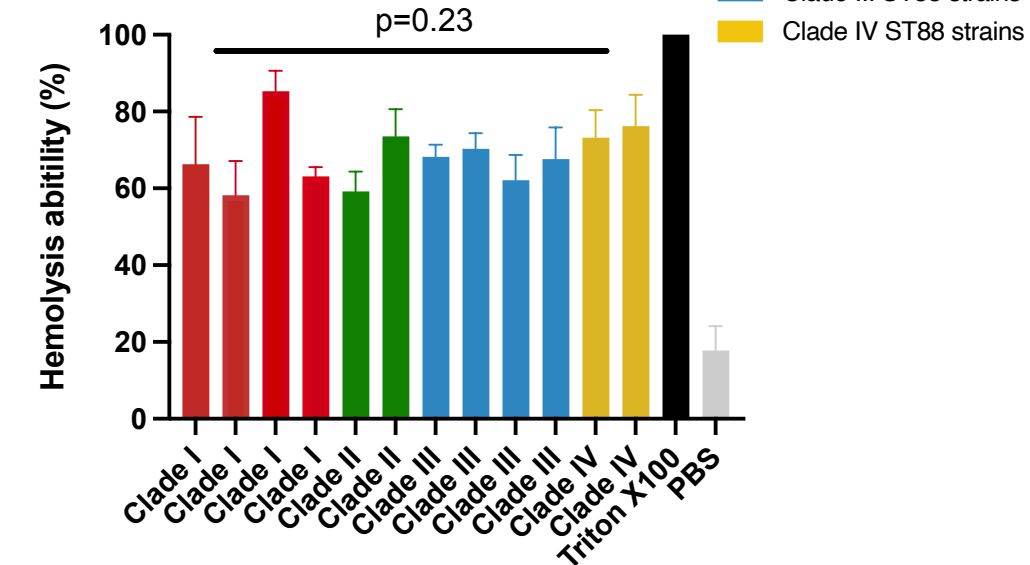

Supplement: Fig. S3 — Comparison of virulence among different clades of ST88 strains. [file msystems.01142-24-s0003.pdf]

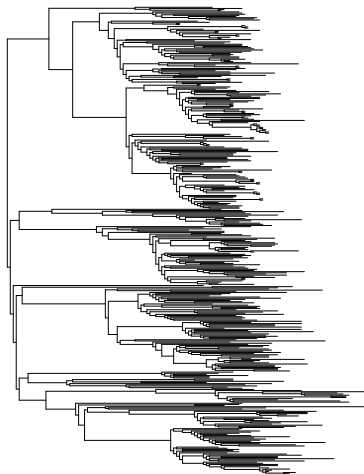

Clade

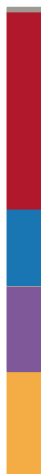

SaPI3

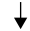

$\Phi$ Sa3

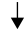

Tn552

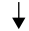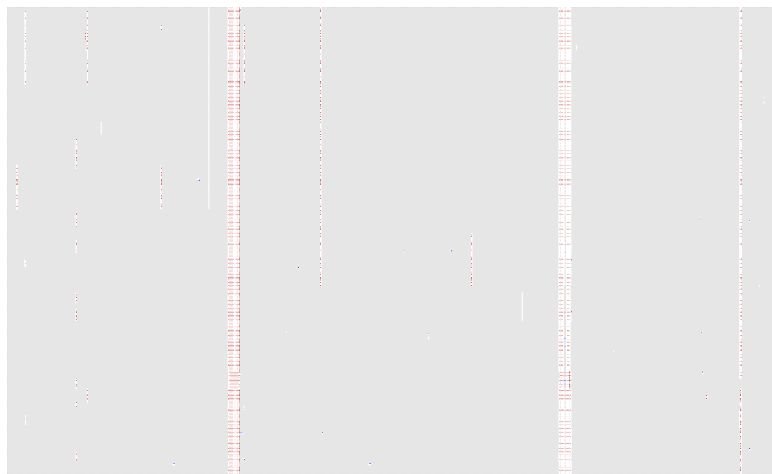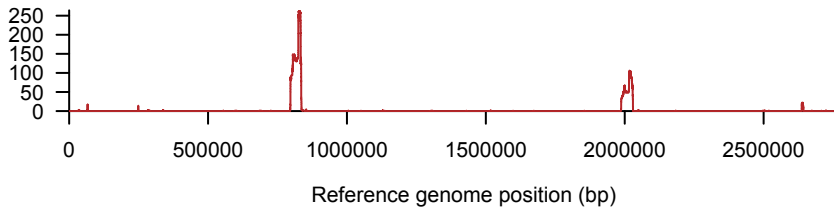

2e-05

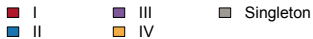

Supplement: Fig. S4 — Recombination plot of the ST88 genomes compared with the reference genome (AUS0325). [file msystems.01142-24-s0004.pdf]

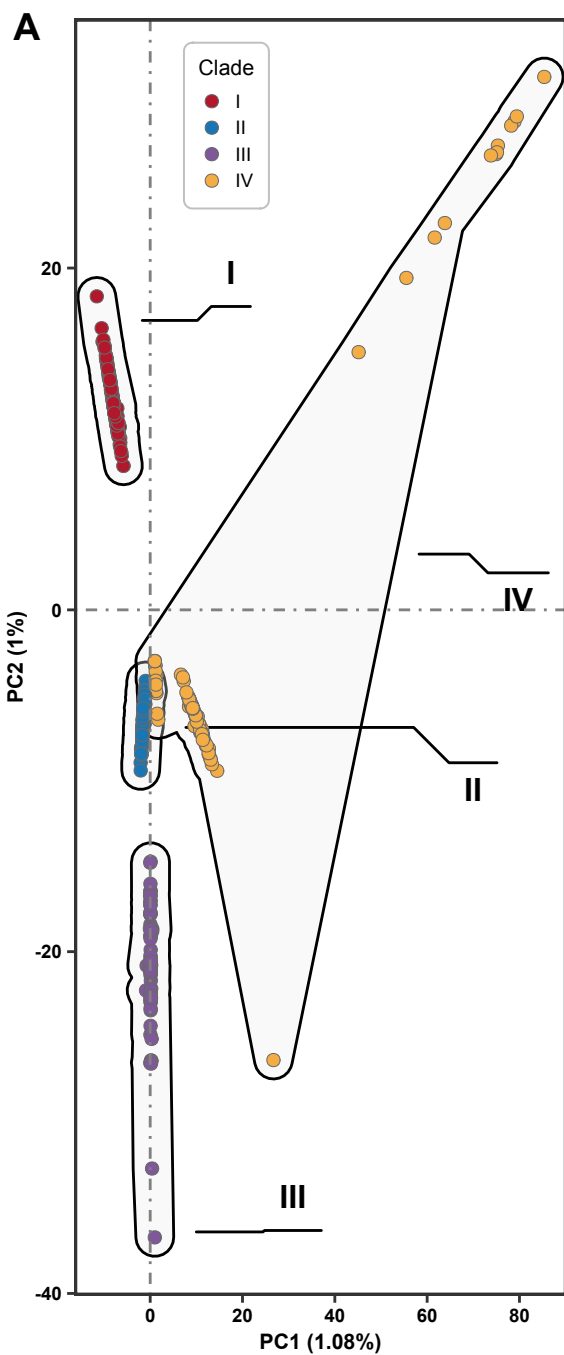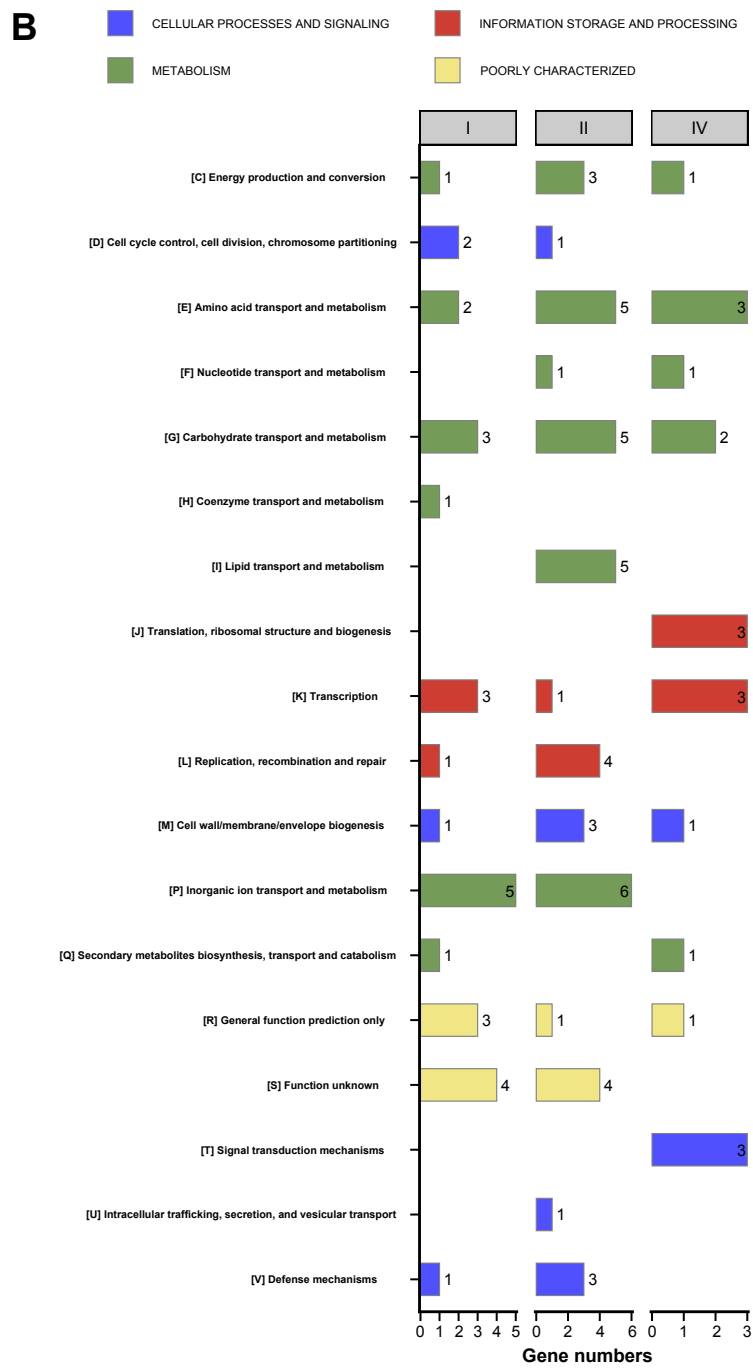

Supplement: Fig. S5 — Core SNP diversity among ST88 clades. [file msystems.01142-24-s0005.pdf]
