# Supplementary material for: Dissecting the genetic features and evolution of Staphylococcus aureus sequence type 88: a global perspective
Source: mSystems. 2024 Nov 12;9(12):e01142-24. doi: 10.1128/msystems.01142-24 (PMC11651095; doi:10.1128/msystems.01142-24)
Supplement: Legends — Fig. S1 to S5 legends. [file msystems.01142-24-s0006.docx]

***Figure S1. Relationships among 130 ST88 isolates from China in our own collection.*** (A) Phylogenetic tree of the 130 ST88 isolates, with tip colors representing the provinces of isolation. (B) Geographic distribution of the isolates, with MRSA and MSSA indicated by red and black partitions within each circle, respectively. (C) Minimum spanning tree (MST) of the ST88 isolates. Each circle represents a ST88 isolate, with colors assigned according to geographic origin. The numbers along the connecting lines denote the number of SNPs in pairwise comparisons. Transmission clusters are highlighted with a red dotted line, defined by a threshold of 23 SNPs.

***Figure S2. Comparison of sraP (A), sasC (B) and φSa3 (C) between ST88 strains in each clade.***

Genes are indicated by blocks (A & B) or arrowed boxes (C), and colored based on domain/gene function classification.

***Figure S3. Comparison of virulence among different clades of ST88 strains.***

(A), (B): Comparison of the ability of different ST88 clades to induce cytotoxicity in mouse RAW264.7 cells. Levels of IL-6 and TNF-α in the cell culture supernatants were measured by enzyme-linked immunosorbent assay (ELISA) after 6 hours of infection. Phosphate-buffered saline (PBS) was used as a negative control. (C): Comparison of the hemolytic activity of different ST88 clades. Experiments were repeated at least three times. Error bars represent the standard deviation (SD). Statistical significance was determined using Student's t-test; ns indicates no statistical significance.

***Figure S4. Recombination plot of the ST88 genomes compared to the reference genome (AUS0325).***

Recombination events detected at non-terminal nodes on the phylogenetic tree are indicated in red, while events that occur exclusively within individual isolates are shown in blue. The line graph below the heatmap displays the frequency of recombination events at corresponding positions in the reference genome. Major recombination hotspots are annotated with arrows and text above the heatmap.

***Figure S5. Core SNP diversity among ST88 clades.***

(A) PCA based on the core SNP matrix of 405 isolates, where each circle represents an isolate, colored according to its clade. (B) COG function classiﬁcation of genes with clade-specific nonsynonymous SNPs.
